# A Multitask Active Learning Framework with Probabilistic Modeling for Multi-Species Acute Toxicity Prediction

**DOI:** 10.3390/molecules31071144

**Published:** 2026-03-30

**Authors:** Tianyu Han, Jingjing Wang, Yanpeng Zhao, Ying Lin, Lu Yu, Song He, Peng Zan, Xiaochen Bo

**Affiliations:** 1 Shanghai Key Laboratory of Power Station Automation Technology, School of Mechatronics Engineering and Automation, Shanghai University, Shanghai 200444, China; teeyohan@163.com (T.H.); 18993412881@163.com (Y.L.); yulu1166@shu.edu.cn (L.Y.); 2 School of Environmental and Chemical Engineering, Shanghai University, Shanghai 200444, China; wangjingjing87@shu.edu.cn; 3 School of Medicine, Shanghai University, Shanghai 200444, China; zhaoyanpeng@shu.edu.cn; 4 Academy of Military Medical Sciences, Beijing 100850, China

**Keywords:** acute toxicity prediction, multi-task learning, active learning, probabilistic model

## Abstract

Predicting acute toxicity across species is essential for early-stage drug safety evaluation. While recent efforts have primarily focused on improving predictive accuracy, they often fail to address two critical issues: the substantial divergence in toxicity mechanisms among different species, and the inherent noise present in experimental data. To bridge this gap, we introduce a Probabilistic Multitask Active Learning (PMAL) framework for multi-species acute toxicity prediction. Our framework integrates two key modules: a Probabilistic Multitask Learning (PML) component which jointly models the predictive distributions of multiple toxicity endpoints from a probabilistic viewpoint, and an Uncertainty-based Active Learning (UAL) component which strategically selects the most informative compounds for experimental annotation based on predictive uncertainty. Empirical evaluations demonstrate that PMAL surpasses state-of-the-art methods and is capable of providing well-calibrated uncertainty estimates for small molecules across diverse toxicity endpoints. Beyond advancing multi-species toxicity prediction, the core design principles of PMAL offer a generalizable paradigm for learning in noisy multi-task environments.

## 1. Introduction

Acute toxicity is defined as adverse physiological effects arising from a single or repeated exposure to exogenous chemical compounds within a 24-h period in humans or experimental animals. Notably, toxicity-related attrition accounts for over 30% of candidate drug failures during early-stage pharmaceutical development [1,2], underscoring the critical need for reliable in silico acute toxicity prediction [3,4]. Robust predictive models enable efficient prioritization of compound libraries by identifying high-risk molecules prior to costly and time-consuming in vitro and in vivo assays, thereby reducing development costs, accelerating lead optimization, and improving overall attrition rates. Nevertheless, accurate and generalizable acute toxicity prediction remains challenging due to data scarcity across species, assay variability, structural diversity of chemical space, and intrinsic biological complexity.

Small-molecule toxicity frequently exhibits pronounced species specificity, i.e., marked inter-species variation in toxicological response profiles, including differences in target engagement, metabolic activation, detoxification pathways, and downstream physiological outcomes. Such variability critically undermines the reliability of safety extrapolation from preclinical animal studies to human clinical trials and imposes stringent demands on the robustness and transferability of computational toxicity prediction models [5]. Consequently, conventional Single-Task Learning (STL) approaches (e.g., training independent models for each species) are inherently limited in their capacity for cross-species generalization. To address this limitation, Multi-Task Learning (MTL) has been increasingly adopted in toxicological modeling. By jointly optimizing predictions across multiple species-specific toxicity endpoints under a shared representational framework, MTL improves predictive accuracy, calibration, and out-of-distribution generalization.

Current MTL approaches [6] fall broadly into two methodological paradigms: (i) architectural design, where task relationships are encoded via shared and task-specific neural modules; and (ii) optimization strategy, where training dynamics are controlled to balance competing objectives. Architectural methods commonly employ encoder–decoder frameworks in which a shared encoder extracts cross-species invariant representations while task-specific decoders model species-dependent toxicological responses. In contrast, optimization-based methods explicitly manage gradient conflicts or loss imbalances to promote equitable learning across toxicity endpoints. Despite these advances, a fundamental bottleneck persists in that MTL performance remains critically constrained by the scarcity and uneven distribution of high-quality and experimentally validated toxicity labels across species, limiting both model calibration and out-of-distribution generalization.

Generating high-quality toxicity annotations requires resource-intensive biological assays, entailing substantial financial cost, prolonged experimental timelines, and rigorous ethical oversight. Consequently, large-scale, consistently curated, and species-balanced toxicity datasets remain scarce. To mitigate this data bottleneck, Active Learning (AL) has emerged as a principled strategy for synergistic model–data co-development in toxicology. Rather than random or passive sampling, AL iteratively selects compounds with maximal information for targeted experimental validation, thereby maximizing knowledge gain per assay and enabling high-accuracy modeling with significantly reduced labeling effort.

Current AL selection strategies [7] fall into two principal categories: uncertainty-based and diversity-based. Uncertainty-based methods prioritize compounds for which the model exhibits high predictive uncertainty, under the premise that such instances are most likely to refine decision boundaries and reveal model deficiencies. In contrast, diversity-based methods seek to maximize coverage of the underlying chemical space by selecting samples that are both representative and minimally redundant relative to the already-labeled set, ensuring robust generalization. A critical limitation of both paradigms is their reliance on deterministic deep learning models, which fail to distinguish between epistemic uncertainty (model ignorance, reducible via data) and aleatoric uncertainty (inherent noise, e.g., assay variability, inter-laboratory discrepancies, or biological stochasticity). Consequently, these approaches cannot reliably quantify or correct for toxicity annotation bias arising from heterogeneous experimental conditions.

In real-world small-molecule toxicity assessment, experimental measurements for the same compound at a given toxicity endpoint frequently exhibit inter-laboratory and intra-assay variability [8]. Deterministic models produce point predictions without uncertainty quantification, making them inherently vulnerable to such label noise and consequently undermining their reliability and credibility. To address this fundamental limitation, probabilistic modeling has emerged as a principled framework for robust toxicity prediction. Unlike deterministic models, probabilistic models explicitly parameterize the full predictive distribution, which not only mitigates bias induced by noisy or inconsistent toxicity labels but also supports calibrated and risk-aware inference for decision-critical toxicological assessment.

Existing probabilistic modeling approaches (e.g., Monte Carlo dropout [9]) rely on repeated stochastic forward passes to approximate the predictive posterior distribution. This “posterior sampling” paradigm, while conceptually straightforward, imposes substantial computational overhead and increases model deployment complexity, particularly in resource-constrained or real-time settings. To overcome these limitations, we propose a direct parameterization framework that explicitly learns the parameters of the predictive distribution (e.g., mixture weights, means, and variances) in a single forward pass, thereby enabling efficient, scalable, and fully differentiable uncertainty quantification. Furthermore, we adopt Gaussian Mixture Models (GMMs) [10] as the parametric form of the predictive distribution. GMMs offer superior flexibility for modeling heterogeneous acute toxicity data, naturally accommodating both irreducible experimental noise and systematic mechanistic differences across species. By explicitly representing multi-task uncertainty structures, our GMM-based formulation yields not only better-calibrated prediction intervals but also interpretable and mechanism-aware uncertainty estimates.

**Figure 1:**
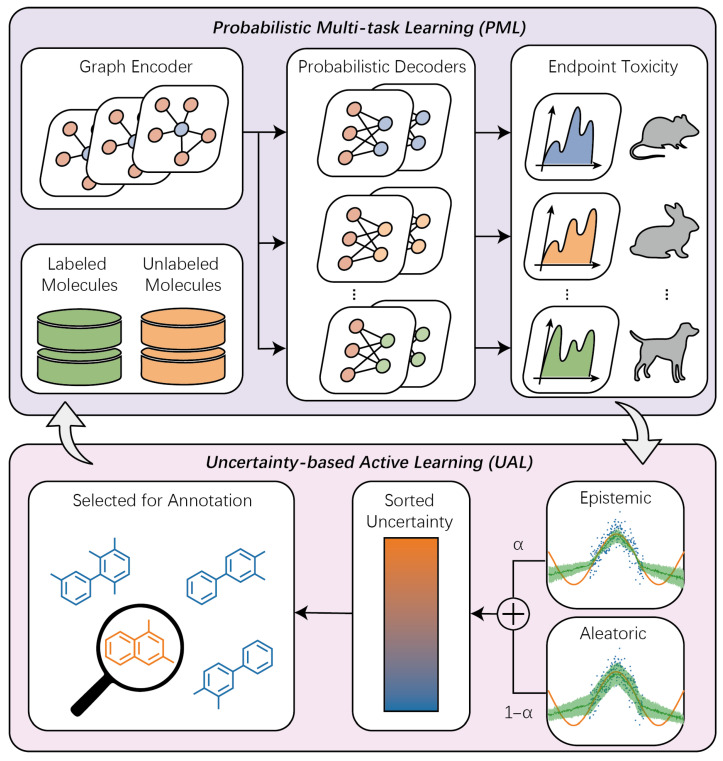
The Probabilistic Multitask Active Learning (PMAL) framework comprises two components: (i) Probabilistic Multitask Learning (PML), which jointly models all toxicity endpoints using a shared representation encoder and endpoint-specific probabilistic decoders to output calibrated predictive distributions; and (ii) Uncertainty-based Active Learning (UAL), which leverages both epistemic and aleatoric uncertainty estimates from PML’s outputs to guide iterative information-maximizing compound selection for experimental validation-aware model calibration.

Building on the foregoing analysis, we propose the Probabilistic Multitask Active Learning (PMAL) framework for multi-species acute toxicity prediction (Figure 1). The core model employs a Graph Neural Network (GNN)-based encoder–decoder architecture in which a shared graph encoder learns invariant molecular representations across species and toxicity endpoints while task-specific probabilistic decoders parameterize predictive distributions. Critically, PMAL jointly quantifies epistemic uncertainty and aleatoric uncertainty for each unlabeled compound. These uncertainties are weighted and aggregated into a unified query score. Compounds are then ranked by descending query score and the top-k compounds are selected for targeted experimental validation. Newly acquired labels are incrementally integrated into the training set in order to refine both representation learning and uncertainty calibration, enabling iterative improvement in predictive accuracy, robustness, and decision-relevant uncertainty quantification.

The contributions of this work are as follows:

- We introduce the Probabilistic Multitask Active Learning (PMAL) framework tailored for multi-species acute toxicity prediction, which unifies multi-task learning and active learning to jointly address data scarcity and label noise in toxicological modeling.
- We formulate a probabilistic multi-task model that explicitly accounts for both epistemic and aleatoric uncertainty; leveraging this dual uncertainty quantification, we further design an active learning query strategy that prioritizes high-uncertainty compounds for experimental labeling.
- We conduct comprehensive experiments across 59 diverse acute toxicity endpoints spanning multiple species, demonstrating consistent improvements over established state-of-the-art methods under realistic low-labeling-budget settings.

## 2. Related Work

Early toxicity research predominantly employed statistical learning methods to develop Quantitative Structure–Activity Relationship (QSAR) models such as linear regression [11], support vector machine [12], and random forest [13]. The advent of deep learning catalyzed a paradigm shift, with deep neural networks now routinely integrated into QSAR modeling. A representative advance is the Deep Learning Consensus Architecture (DLCA) [14], which synergistically unifies consensus modeling and MTL to scale QSAR prediction across diverse endpoints. Subsequent studies [15,16] systematically benchmarked multi-task toxicity prediction across broad chemical spaces, varying both molecular descriptors and algorithmic frameworks. Crucially, progress has accelerated not only in model sophistication but also in data infrastructure and biological relevance; recent efforts have established large-scale and high-quality toxicity data platforms [17,18], adopted more advanced methods [19], and conducted organ-specific toxicity assessment grounded in human-relevant biology [20].

MTL and AL have advanced rapidly in computer vision and natural language processing, and their adoption in computational toxicology is now accelerating. In MTL, researchers have developed novel neural architectures that jointly predict multiple tasks [21,22,23,24], significantly enhanced by integration with GNNs [25,26,27] to exploit molecular topology for unified structure-aware representation learning. Critically, these efforts confront the persistent challenge of task imbalance, in which divergent loss magnitudes and convergence rates across tasks can induce gradient conflict and degrade shared representation quality. To mitigate this, recent work has introduced principled optimization strategies such as dynamic loss weighting [28,29,30] and gradient surgery techniques that orthogonalize task-specific gradients [31,32,33], leading to improved training stability and cross-task generalization. In AL, the high cost of experimental annotation has driven intensive innovation in query strategies. While uncertainty-based selection remains dominant, e.g., entropy-maximization [34,35,36] or loss-guided identification of ambiguous samples [37,38], diversity-promoting approaches [39,40] are increasingly recognized for their ability to ensure broad coverage of chemical space, enabling more efficient model refinement under stringent labeling budgets.

## 3. Results

### 3.1. Experimental Settings

#### 3.1.1. Dataset

We evaluated the effectiveness of PMAL using the multi-species acute toxicity dataset ToxAcute [41]. This dataset comprises 120,384 experimentally measured data points across 59 distinct toxicity endpoints, each defined by a unique combination of species (mouse, rat, mammal, guinea pig, rabbit, dog, cat, wild bird, quail, duck, chicken, frog, and human), administration route (intraperitoneal, intravenous, oral, transdermal, subcutaneous, intramuscular, and non-intestinal), and toxicity metric (median lethal dose (LD50), lowest observed lethal dose (LDLo), and lowest observed toxic dose (TDLo)). For each endpoint, we performed scaffold split into training, validation, and test sets at an 8:1:1 ratio to ensure structural independence. To stabilize model optimization and improve numerical conditioning, all dose values were transformed to their negative logarithm in units of mol/kg (i.e., −log(mol/kg)). Under this transformation, higher values correspond to greater toxicity for a given compound–endpoint pair.

#### 3.1.2. Metrics

We evaluate model performance using the Root Mean Squared Error (RMSE) and the Coefficient of Determination (R^2^):

- Root Mean Squared Error (RMSE): Quantifies the average magnitude of prediction errors in the target variable. It is defined as follows:
  (1) $$\mathrm{RMSE}=\sqrt{\frac{1}{n}\sum_{i=1}^{n}{\left(y_{i}-{\hat{y}}_{i}\right)}^{2}},$$
  where *n* is the total number of samples, $y_{i}$ denotes the true value, and ${\hat{y}}_{i}$ denotes the predicted value for the i-th sample.
- Coefficient of Determination (R^2^): Measures the proportion of variance in the observed outcomes explained by the model, computed as follows:
  (2) $$R^{2}=1-\frac{\sum_{i=1}^{n}{\left(y_{i}-{\hat{y}}_{i}\right)}^{2}}{\sum_{i=1}^{n}{\left(y_{i}-\bar{y}\right)}^{2}},$$
  where $\bar{y}=\frac{1}{n}\sum_{i=1}^{n}y_{i}$ is the mean of the observed values.

#### 3.1.3. Training Details

All experiments were conducted on a single NVIDIA RTX 4090 GPU. We implemented the training and inference pipeline using PyTorch 1.8.1 and PyTorch Geometric 2.2.0, and performed molecular processing using RDKit 2022.3.3. Each molecule was represented as an undirected graph in which node features were encoded as a 96-dimensional vector comprising the following: atomic number (44 dimensions), atom degree (17 dimensions), implicit hydrogen count (17 dimensions), valence (17 dimensions), and aromaticity flag (1 dimension). For each endpoint, the AL protocol began with an initial labeled set containing 10% of labeled samples; three iterative rounds followed, each querying the top 10% of unlabeled pool based on annotation utility. Within each round, models were trained for 50 epochs with a batch size of 64, using the Adam optimizer (learning rate 1 × 10^−3^, weight decay 1 × 10^−5^). To ensure robustness, all experiments were repeated three times, and results are reported as mean ± standard deviation.

### 3.2. Comparative Experiments

To evaluate PMAL, we benchmarked it against a comprehensive set of established baselines on the ToxAcute dataset under two learning paradigms: MTL and AL. For MTL, we compared PMAL with three GNN-based approaches (MTGCN [25], MTGAT [26], and MTGIN [27]), each representing distinct architectural inductive biases for molecular representation learning. Under AL, we evaluated PMAL against five representative strategies (Random, Entropy [34], CoreSet [39], ProbCover [40], and TiDAL [38]). Model performance is assessed using both RMSE and R^2^, with each reported value representing the mean across all 59 toxicity endpoints.

#### 3.2.1. Multi-Task Learning Setting

The comparative performance of PMAL against representative MTL methods is summarized in Table 1 and Table 2. As shown in the results, PMAL achieved state-of-the-art performance on 6 out of 14 evaluation tasks (including 13 individual species and their averaged score), surpassing all other MTGNNs by a substantial margin. Moreover, model performance across species exhibited a positive correlation with the volume of available labeled data, confirming the expected data scalability behavior. Notably, PMAL achieved the highest performance among all compared methods on several particularly challenging species (e.g., guinea pig, cat, and frog) which are characterized by limited annotated samples. This consistent superiority on low-resource species strongly supports PMAL’s enhanced generalization capability under small-sample conditions.

#### 3.2.2. Active Learning Setting

The comparative performance of PMAL against representative AL methods is summarized in Table 3 and Table 4. As shown in the results, Entropy underperformed even Random sampling, highlighting its limited utility for acute toxicity prediction in this multi-endpoint setting. In contrast, diversity-based approaches (CoreSet and ProbCover) consistently outperformed uncertainty-based ones (Entropy and TiDAL), indicating that structural coverage of the chemical space is more informative than prediction variance alone for this task. While PMAL exhibited marginally lower initial performance relative to these baselines, it demonstrated consistent improvement across successive AL rounds and ultimately achieved superior final performance. Crucially, PMAL integrates dual-source uncertainty estimation, a capability that is absent in existing diversity-based methods.

### 3.3. Effectiveness Analysis

This section empirically evaluates PMAL along three complementary dimensions: (i) its probabilistic modeling capability, specifically whether the uncertainty estimates it produces are well calibrated and meaningfully reflect true prediction uncertainty; (ii) its MTL efficacy, assessing whether joint optimization yields performance competitive with or superior to that of the STL setting; and (iii) the effectiveness of its active sample annotation, investigating whether the instances selected by PMAL consistently drive measurable improvements in downstream model performance.

#### 3.3.1. Probability Modeling

**Figure 2:**
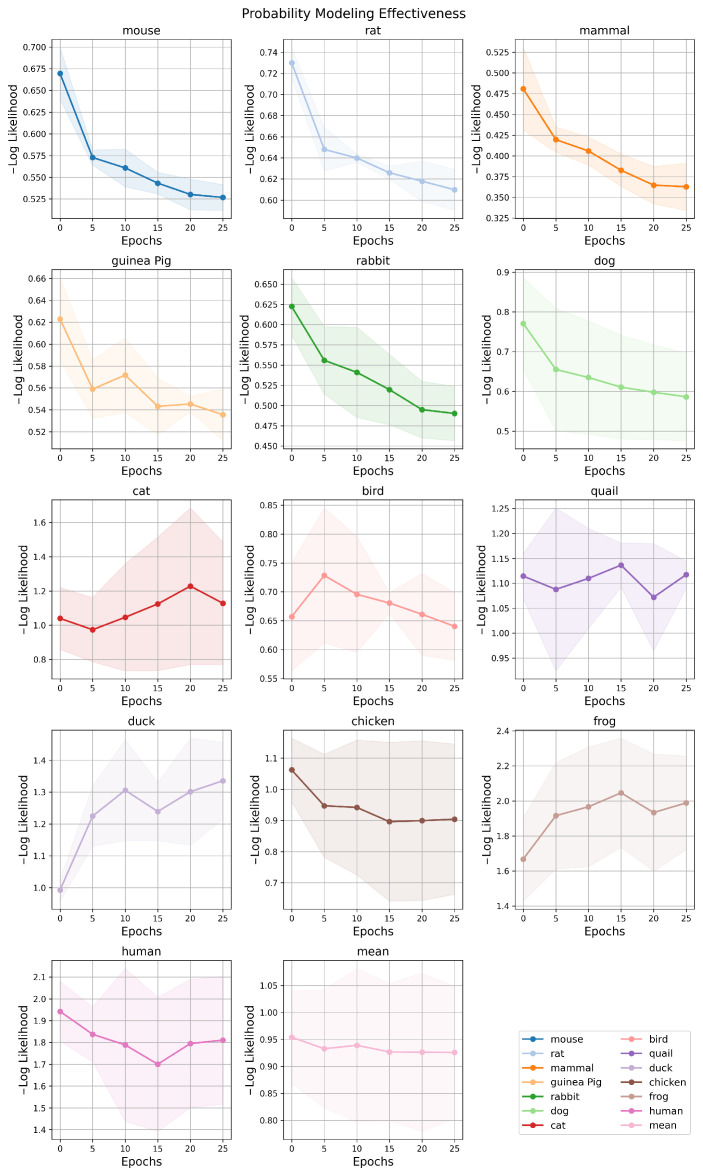
Probability modeling effectiveness analysis. Each subplot displays the evolution of the negative log-likelihood over training epochs, with a distinct color assigned to each species.

To evaluate PMAL’s probabilistic modeling capability across diverse toxicity prediction tasks, we monitored the Negative Log-Likelihood (NLL) over training epochs; the results are presented in Figure 2. For the majority of species, PMAL’s NLL consistently decreases with training progress, demonstrating robust and well-calibrated probabilistic predictions on multi-species acute toxicity data. In contrast, NLL increases for a small subset of species (e.g., duck and frog) indicating task conflict during multi-task optimization. The performance gains on dominant tasks appear to come at the expense of predictive calibration for these underrepresented or structurally dissimilar species. Such tradeoffs reflect a known challenge in MTL, particularly when task objectives exhibit misaligned gradients or imbalanced data coverage. Nevertheless, PMAL achieves superior overall performance compared to competing methods (Table 1 and Table 2), confirming its effectiveness in multi-task uncertainty modeling.

#### 3.3.2. Multi-Task Training

**Figure 3:**
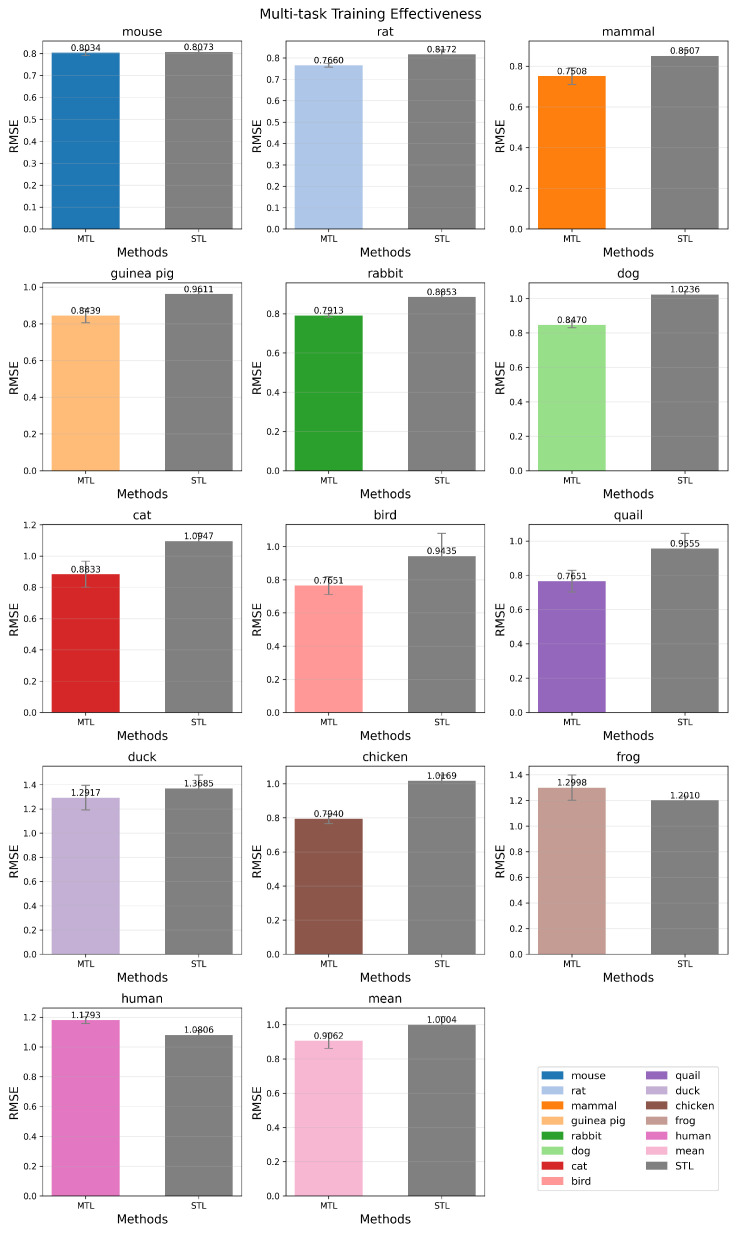
Multi-task integration effectiveness analysis. Each subplot presents a side-by-side comparison of model performance under the MTL and STL paradigms, with a distinct color assigned to each species.

To assess whether multi-task joint training yields superior generalization compared to single-task training, we conducted a per-species side-by-side comparison of RMSE performance, as presented in Figure 3. For most species, the multi-task model achieved significantly lower RMSE than its single-task counterpart, suggesting that acute toxicity profiles across these species share underlying structural or mechanistic regularities. These shared patterns enable effective knowledge transfer and cross-species regularization, thereby improving predictive robustness. Notably, however, the multi-task approach underperformed for a minority of species (e.g., frog and human). This degradation implies limited transferability between certain phylogenetically or physiologically distant species, highlighting a fundamental limitation in domain adaptation: when source and target distributions diverge substantially in feature space or biological mechanism, shared representations may introduce negative transfer. This phenomenon remains an active area of investigation in toxicological modeling and cross-species extrapolation.

#### 3.3.3. Active Annotation

**Figure 4:**
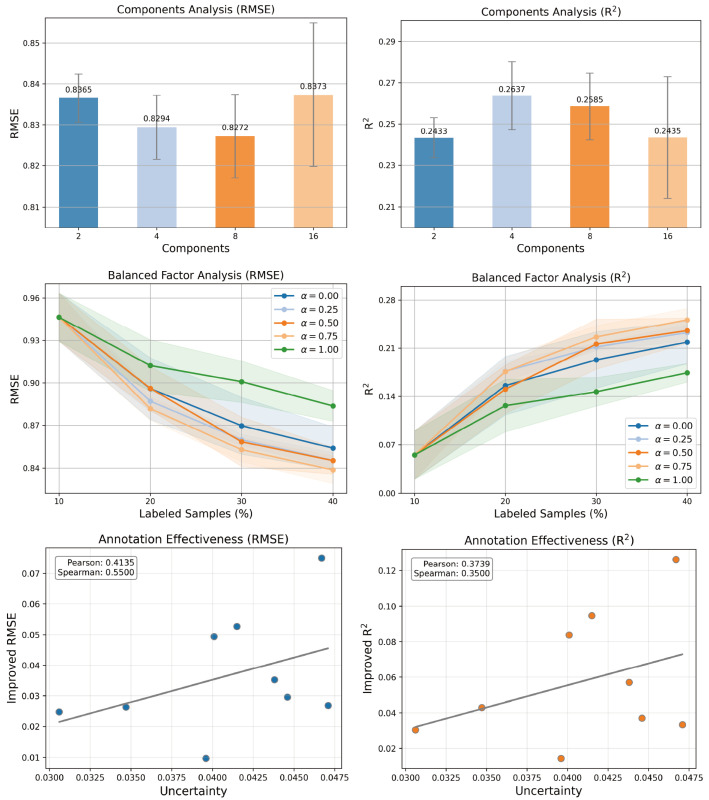
Hyperparameter and active annotation effectiveness analysis. (**Upper**): Component number (*C*) analysis. (**Middle**): Balance factor (α) analysis. (**Lower**): Active annotation effectiveness analysis.

An effective query strategy should prioritize label-beneficial samples, that is, those with annotations that yield the largest marginal gain in model performance, rather than selecting samples based solely on task-irrelevant attributes. As shown in Figure 4 (Lower), we compute both Pearson and Spearman correlation coefficients between the uncertainty and the corresponding marginal performance gain (measured as the decrease/increase in RMSE/R^2^ metric after incorporating each labeled sample into the training set). The resulting coefficients range from 0.3 to 0.6, indicating a statistically meaningful and moderately positive monotonic association. Crucially, this correlation holds across two evaluation metrics, suggesting that higher uncertainty reliably identifies samples with high annotation utility.

### 3.4. Hyperparameter Analysis

This section analyzes two critical hyperparameters in PMAL: the number of components (*C*) in the PML, and the balance factor (α) in the UAL. Specifically, *C* determines the capacity of the Gaussian mixture model to capture uncertainty patterns across acute toxicity endpoints; meanwhile, α governs the tradeoff between epistemic and aleatory uncertainty in the composite uncertainty score.

#### 3.4.1. Number of Components

To investigate the impact of the number of components (*C*) on the capacity of PML, we conducted an analysis over C∈{2,4,8,16}, evaluating mean RMSE and R^2^ across all 59 toxicity endpoints. The results are presented in Figure 4. As shown, the lowest RMSE is achieved at C=8, whereas the highest R^2^ occurs at C=4. Moreover, increasing *C* leads to a monotonic rise in the standard deviation of performance, reflecting growing sensitivity to training instability in mixture models. Given that R^2^ better reflects predictive reliability for multi-endpoint toxicity regression and that C=4 achieves both competitive RMSE and markedly lower variance, we adopt C=4 as the default setting.

#### 3.4.2. Balanced Factor

To assess the influence of the balance factor (α) on the capacity of UAL, we performed an ablation study over α∈{0.0,0.25,0.5,0.75,1.0}, evaluating mean RMSE and R^2^ across all 59 toxicity endpoints (Figure 4). As shown, α=1.0 (i.e., exclusive reliance on epistemic uncertainty) yields the worst performance. This degradation reflects the model’s inability to account for inherent noise within the outcome. Consequently, integrating both uncertainty sources is essential for robust sample selection. Among all configurations, α=0.75 achieves the strongest performance, striking the best empirical tradeoff between error minimization and explained variance. We therefore adopt α=0.75 as the default balance factor.

### 3.5. Visualizations

This section presents a three-fold visualization analysis of PMAL: (i) prediction credibility, (ii) species-level uncertainty distribution, and (iii) species-level embedding distributions. Each aspect is examined in turn through visualizations.

#### 3.5.1. Prediction Visualizations

**Figure 5:**
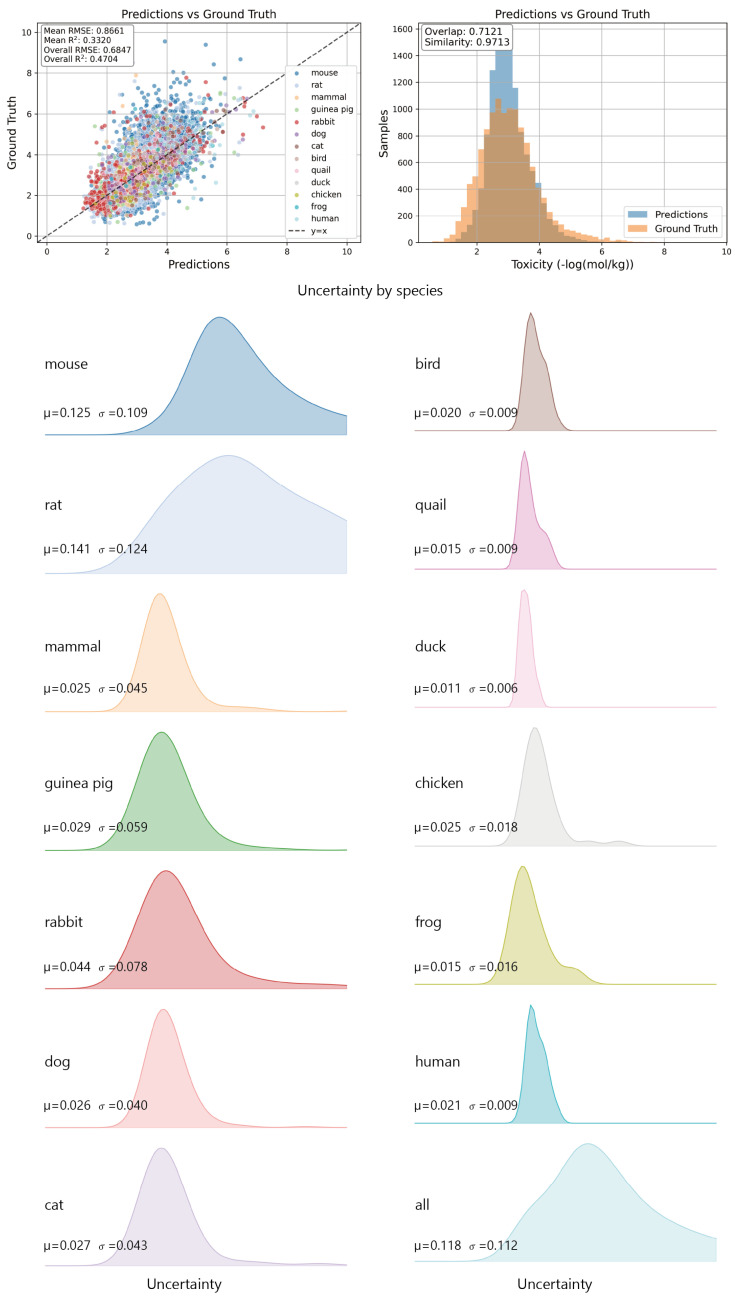
Prediction and uncertainty visualization. (**Upper**): PMAL’s predicted toxicity values (grouped by species). (**Lower**): Species-level uncertainty estimates. Species are distinguished by distinct colors.

This subsection evaluates the credibility of PMAL’s predictions. Figure 5 presents scatter plots (left) and histograms (right) comparing model predictions against ground-truth values across species. As shown in the scatter plot, PMAL predictions exhibit alignment with the y=x line, with overall-level results outperforming species-level results. This gap mainly stems from the highly unbalanced distribution of toxicity data among different species. The histogram confirms strong distributional fidelity: the distributions of the predicted and ground-truth values exhibit high overlap, and their statistical distributions are highly similar.

#### 3.5.2. Uncertainty Visualizations

This subsection characterizes the species-level uncertainty distribution estimated by PMAL. For each species, we aggregate uncertainty scores across all test samples and visualize the resulting distributions using Gaussian Kernel Density Estimation (KDE) [42] (Figure 5). We observe that uncertainty distributions correlate with both phylogenetic proximity and data coverage. Specifically, mouse and rat exhibit elevated mean uncertainty and broader dispersion, consistent with their extensive chemical space coverage. In contrast, bird and quail show nearly identical KDE curves, reflecting shared avian physiology and comparable assay protocols.

#### 3.5.3. Representation Visualizations

**Figure 6:**
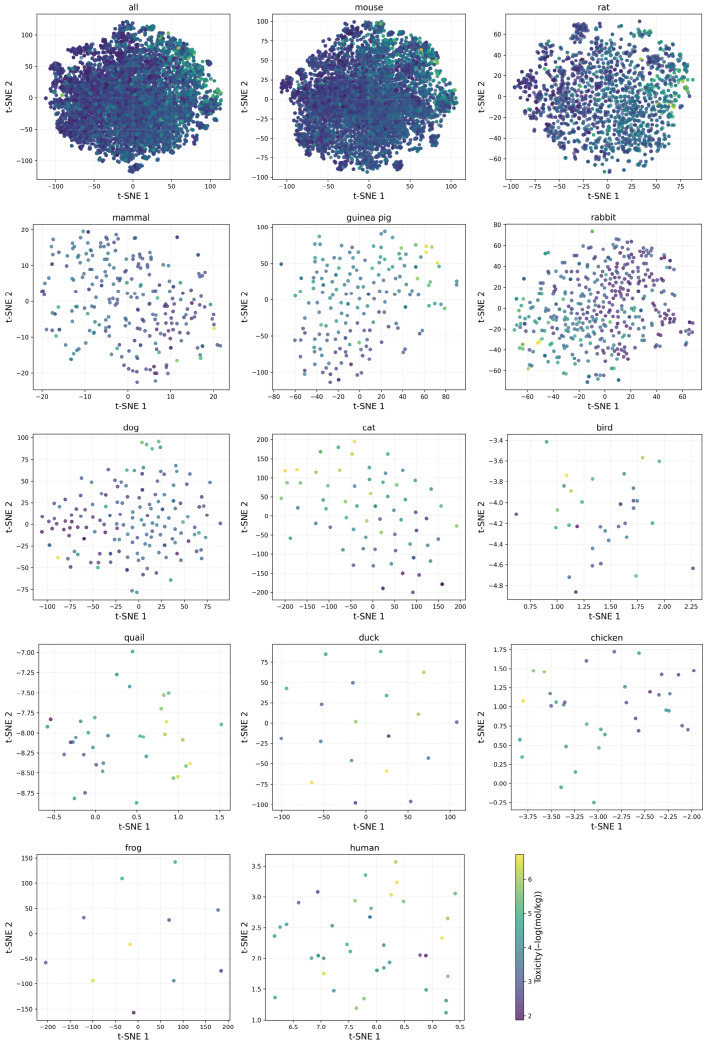
Representation visualizations, showing t-SNE visualization of PMAL representations across species. Toxicity values are encoded via a continuous color; darker hues correspond to lower toxicity, while lighter hues correspond to higher toxicity.

This subsection examines the species-level embedding distributions learned by PMAL. Figure 6 shows t-SNE projections [43] of embeddings for thirteen species. The projections reveal pronounced inter-species divergence in latent geometry: high-sample species (e.g., mouse and rat) exhibit smooth, dose-ordered manifolds; by contrast, low-sample species (e.g., duck and frog) yield fragmented isotropic clouds with no discernible dose gradient, reflecting insufficient capacity for structured embedding formation. This stark contrast underscores a fundamental limitation in which representation quality in PMAL remains strongly contingent on per-species data volume, highlighting the persistent challenge of few-shot toxicological generalization.

## 4. Method

### 4.1. Notations

We formalize each acute toxicity endpoint (e.g., specific biological species, route of administration, and toxicity metric) as an individual learning task. Let $T=\{T_{1},T_{2},\ldots ,T_{K}\}$ denote the set of *K* such tasks. For each task $T_{k}$, we define three sets: a labeled set $X_{k}^{\mathrm{label}}$, an unlabeled set $X_{k}^{\mathrm{unlabel}}$, and a label set $Y_{k}$. Every labeled instance $x\in X_{k}^{\mathrm{label}}$ is associated with a ground-truth label $y\in Y_{k}$; in contrast, instances $x\in X_{k}^{\mathrm{unlabel}}$ lack corresponding annotations. Crucially, each molecule *x* is represented as an undirected graph G={V,E} in which vertices *V* correspond to atoms and edges *E* encode chemical bonds between them.

The MTL model *f* adopts an encoder–decoder architecture comprising a shared encoder $f^{\mathrm{en}}$ and *K* task-specific decoders ${\left\{f_{k}^{\mathrm{de}}\right\}}_{k=1}^{K}$. Its learnable parameters are partitioned into two disjoint sets: a shared parameter $\theta_{s}$ governing the encoder $f^{\mathrm{en}}$, and task-specific parameters ${\left\{\theta_{k}\right\}}_{k=1}^{K}$, each associated with decoder $f_{k}^{\mathrm{de}}$. Given a molecule *x*, the encoder first computes a task-invariant latent representation $z=f^{\mathrm{en}}\left(x;\theta_{s}\right)$. This shared representation is then fed into the k-th decoder to yield the task-specific prediction ${\hat{y}}_{k}=f_{k}^{\mathrm{de}}\left(z;\theta_{k}\right)$. The overall objective is to jointly optimize all parameters by minimizing the weighted sum of expected task losses:

(3) $$\min_{\theta_{s},\theta_{1},\ldots ,\theta_{K}}\sum_{k=1}^{K}E_{\left\{x,y\right\}∼\left\{X_{k}^{\mathrm{label}},Y_{k}\right\}}\left[L_{k}\left({\hat{y}}_{k},y_{k}\right)\right],$$

where $L_{k}$ is the loss function of task $T_{k}$.

The AL is formulated as an iterative refinement process. At iteration *t*, the current multi-task model *f* scores every unlabeled molecule $x\in X^{\mathrm{unlabel}}$ according to a labeling utility metric. The top-B samples with the highest utility are selected to make up the query set $X_{t}^{\mathrm{query}}$. The labeled and unlabeled sets are then updated deterministically:

(4) $$X_{t+1}^{\mathrm{label}}=X_{t}^{\mathrm{label}}\cup X_{t}^{\mathrm{query}},$$

(5) $$X_{t+1}^{\mathrm{unlabel}}=X_{t}^{\mathrm{unlabel}}\setminus X_{t}^{\mathrm{query}},$$

with this cycle repeating until either a predefined iterations is reached or the annotation budget is fully consumed.

### 4.2. PMAL Framework

This paper introduces PMAL, a probabilistic multi-task active learning framework for multi-species acute toxicity prediction, illustrated in Figure 1. PMAL integrates two synergistic components: (i) Probabilistic Multitask Learning (PML), which employs a shared graph encoder to extract invariant molecular representations and task-specific probabilistic decoders to model the full predictive distribution of each toxicity endpoint; and (ii) Uncertainty-based Active Learning (UAL), which jointly quantifies epistemic uncertainty and aleatoric uncertainty and fuses them into a calibrated overall uncertainty score. The active selection module then prioritizes unlabeled molecules by descending overall uncertainty and queries the top-B instances for expert annotation. By iteratively acquiring maximally informative samples under strict annotation budgets, PMAL achieves significant reductions in labeling effort while consistently improving out-of-distribution generalization across diverse species and endpoints.

### 4.3. Probabilistic Multi-Task Learning

PML establishes a principled graph-to-distribution mapping via a shared graph encoder and task-specific probabilistic decoders. The encoder extracts invariant molecular representations from the input graph, while each decoder transforms these representations into a well-calibrated predictive distribution. Critically, this architecture yields not only probabilistic predictions but also intrinsic quantifiable uncertainty estimates (e.g., epistemic and aleatoric uncertainty) directly encoded in the output distribution. As such, it provides a theoretically grounded and empirically actionable uncertainty signal that serves as the essential input to UAL.

The shared graph encoder consists of *L* consecutive Graph Convolutional Network (GCN) layers. Given a molecular graph G={V,E}, it computes a molecule-level embedding *z* via iterative message passing across nodes followed by a Readout operation. Specifically, at layer l+1, the embedding $h_{v}^{\left(l+1\right)}$ of node v∈V is updated as follows:

(6) $$h_{v}^{\left(l+1\right)}=\sigma \left(\sum_{u\in N(v)\cup \{v\}}\frac{1}{\sqrt{\deg(v)\deg(u)}}h_{u}^{\left(l\right)}\theta_{s}^{\left(l\right)}\right),$$

where N(·) denotes the first-order neighborhood, deg(·) is the degree, $h_{u}^{\left(l\right)}$ is the embedding of node *u*, $\theta_{s}^{\left(l\right)}$ is the learnable weight matrix, and σ(·) is the activation function. After *L* layers of message passing, the final node embeddings $h_{v}^{\left(L\right)}$ are aggregated into a fixed-size molecular representation

(7) $$z=\mathrm{READOUT}\left(h_{v}^{\left(L\right)}|v\in V\right),$$

with this embedding *z* serving as the input to each task-specific probabilistic decoder.

Each task-specific probabilistic decoder comprises a Multi-Layer Perceptron (MLP) that maps the shared molecular embedding *z* to the parameters of a C-component Gaussian Mixture Model (GMM). For regression endpoints, the conditional distribution is defined as

(8) $$p\left({\hat{y}}_{c}|z\right)=N\left({\hat{y}}_{c}|\mu_{c}\left(z\right),\sigma_{c}^{2}\left(z\right)\right),$$

where $\mu_{c}\left(\cdot \right)$ and $\sigma_{c}^{2}\left(\cdot \right)$ respectively denote the mean and variance and ${\hat{y}}_{c}$ denotes the component-wise prediction. For classification endpoints, we adopt a stochastic softmax formulation grounded in the reparameterization trick:

(9) $$p\left({\hat{y}}_{c}|z\right)=\mathrm{Softmax}\left(\mu_{c}\left(z\right)+\sqrt{\sigma_{c}^{2}\left(z\right)}\cdot \gamma \right),$$

where γ follows the standard Gaussian distribution. The loss for the k-th toxicity endpoint is defined as

(10) $$L_{k}=-\sum_{c=1}^{C}\pi_{c}\left(z\right)\log p\left({\hat{y}}_{c}|z\right),$$

with $\pi_{c}\left(\cdot \right)$ denoting the mixture weight subject to $\Sigma_{c=1}^{C}\pi_{c}=1$.

### 4.4. Uncertainty-Based Active Learning

Upon completion of PML training, the model’s learned probabilistic outputs provide a principled basis for quantifying sample-level uncertainty [44]. Specifically, uncertainty is decomposed into the two components of epistemic uncertainty and aleatoric uncertainty. Such decomposition enables targeted selection of highly informative samples while explicitly distinguishing between uncertainty that is reducible through labeling (epistemic) and uncertainty that is inherent to the measurement process (aleatoric).

For regression endpoints, aleatoric uncertainty is computed as the mixture-weighted average of component-wise variances, while epistemic uncertainty is quantified as the mixture-weighted variance of the differences between the component-wise means and overall-wise mean:

(11) $$U^{\mathrm{al}}=\sum_{c=1}^{C}\pi_{c}\cdot \sigma_{c}^{2},$$

(12) $$U^{\mathrm{ep}}=\sum_{c=1}^{C}\pi_{c}\cdot ∥\mu_{c}-\sum_{c=1}^{C}\pi_{c}\cdot \mu_{c}{∥}^{2}.$$

For classification endpoints, we estimate aleatoric and epistemic uncertainty via Monte Carlo (MC) sampling over the learned Gaussian mixture model. First, a component is sampled based on the mixed weights, then an independent sample is drawn from the corresponding predictive distribution of that component. This process is repeated *T* times to obtain the predictive probability sequence ${\left\{p_{t}\right\}}_{t=1}^{T}$. Aleatory uncertainty is expressed as the expectation of the predictive entropy of the sampling sequence, and epistemic uncertainty can be obtained by subtracting aleatory uncertainty from the total uncertainty:

(13) $$U^{\mathrm{al}}=\frac{1}{T}\sum_{t=1}^{T}\left[-p_{t}\log p_{t}-\left(1-p_{t}\right)\log\left(1-p_{t}\right)\right],$$

(14) $$U^{\mathrm{ep}}=U^{\mathrm{total}}-U^{\mathrm{al}},$$

where $U^{\mathrm{total}}=-\bar{p}\log\bar{p}-\left(1-\bar{p}\right)\log\left(1-\bar{p}\right)$ is the total uncertainty, reflecting the model’s overall confidence in the final class assignment, and $\bar{p}=\frac{1}{T}\Sigma_{t=1}^{T}p_{t}$ is the average of multiple sampling prediction probabilities.

Finally, we define the annotation utility of each unlabeled sample as a combination of the epistemic and aleatoric uncertainties:

(15) $$U=\alpha \cdot U^{\mathrm{ep}}+\left(1-\alpha \right)\cdot U^{\mathrm{al}},$$

where higher values of U indicate greater informativeness. The unlabeled pool is then sorted in descending order and the top-B samples are selected for expert annotation.

## 5. Conclusions

In this study, we introduce Probabilistic Multitask Active Learning (PMAL), a unified framework designed to tackle two fundamental challenges in multi-species acute toxicity prediction: (i) pronounced interspecies divergence in toxicity response patterns, and (ii) irreducible experimental noise inherent to biological assays. PMAL synergistically integrates Probabilistic Multitask Learning (PML) and Uncertainty-based Active Learning (UAL). PML jointly models the predictive distributions across 59 toxicity endpoints, while UAL leverages this calibrated uncertainty to iteratively prioritize compounds with maximal expected information gain for experimental validation. Empirical evaluation demonstrates that PMAL achieves state-of-the-art prediction accuracy and reduces required experimental effort. Critically, the PMAL paradigm is broadly applicable to any domain in which label scarcity, measurement noise, and cross-task structure coexist, including environmental risk assessment and preclinical safety profiling.

## Figures and Tables

**Table 1:** RMSE performance comparison under MTL settings. The best result is highlighted in bold and the second-best result is underlined.

Species	Labels	MTGCN [25]	MTGAT [26]	MTGIN [27]	PMAL
Mouse	86,231	**0.7795 ± 0.0204**	0.7818 ± 0.0218	0.7825 ± 0.0103	0.7924 ± 0.0131
Rat	21,749	**0.7255 ± 0.0089**	0.7359 ± 0.0196	0.7356 ± 0.0157	0.7559 ± 0.0088
Mammal	2453	0.7132 ± 0.0094	0.7114 ± 0.0273	**0.6987 ± 0.0079**	0.7062 ± 0.0412
Guinea Pig	1743	0.9406 ± 0.0210	0.9293 ± 0.0117	0.9072 ± 0.0118	**0.8091 ± 0.0385**
Rabbit	4283	**0.7138 ± 0.0109**	0.7352 ± 0.0209	0.7187 ± 0.0169	0.7807 ± 0.0092
Dog	1534	0.7698 ± 0.0361	**0.7639 ± 0.0241**	0.7910 ± 0.0253	0.8361 ± 0.0165
Cat	693	1.1136 ± 0.0349	1.1623 ± 0.0037	1.0830 ± 0.0584	**0.8833 ± 0.0828**
Bird	329	**0.6364 ± 0.0342**	0.6654 ± 0.0307	0.6689 ± 0.0490	0.7325 ± 0.0539
Quail	349	0.7651 ± 0.0730	0.7605 ± 0.0157	0.7428 ± 0.0490	**0.7026 ± 0.0635**
Duck	186	1.1770 ± 0.0295	**1.1528 ± 0.0462**	1.1830 ± 0.1293	1.1836 ± 0.1012
Chicken	347	0.8196 ± 0.0594	0.9200 ± 0.0151	0.8702 ± 0.0574	**0.7940 ± 0.0280**
Frog	106	1.2954 ± 0.1203	1.2520 ± 0.0544	1.3084 ± 0.0585	**1.2309 ± 0.0977**
Human	381	1.0756 ± 0.0909	**1.0534 ± 0.0590**	1.1105 ± 0.0325	1.1618 ± 0.0224
Mean	-	0.8865 ± 0.0422	0.8941 ± 0.0269	0.8924 ± 0.0402	**0.8745 ± 0.0444**

**Table 2:** R^2^ performance comparison under MTL settings. The best result is highlighted in bold and the second-best result is underlined.

Species	Labels	MTGCN [25]	MTGAT [26]	MTGIN [27]	PMAL
Mouse	86,231	**0.3059 ± 0.0368**	0.2825 ± 0.0369	0.2913 ± 0.0165	0.2828 ± 0.0179
Rat	21,749	**0.3879 ± 0.0182**	0.3824 ± 0.0316	0.3787 ± 0.0251	0.3506 ± 0.0150
Mammal	2453	0.1885 ± 0.0310	0.1961 ± 0.0677	0.2265 ± 0.0226	**0.2328 ± 0.0916**
Guinea Pig	1743	−0.1206 ± 0.0767	−0.0297 ± 0.0565	0.0436 ± 0.0472	**0.3235 ± 0.0739**
Rabbit	4283	0.3645 ± 0.0451	0.3325 ± 0.0276	**0.3774 ± 0.0031**	0.2685 ± 0.0480
Dog	1534	0.4616 ± 0.0567	**0.4658 ± 0.0333**	0.4330 ± 0.0323	0.3520 ± 0.0191
Cat	693	−0.0676 ± 0.0717	−0.1395 ± 0.0180	−0.0031 ± 0.1387	**0.3860 ± 0.1081**
Bird	329	**0.1811 ± 0.0891**	0.1052 ± 0.0815	0.0939 ± 0.1336	−0.0828 ± 0.1698
Quail	349	0.3744 ± 0.1157	0.3856 ± 0.0252	0.4122 ± 0.0760	**0.4757 ± 0.1033**
Duck	186	0.3352 ± 0.0335	**0.3618 ± 0.0516**	0.3233 ± 0.1425	0.3279 ± 0.1243
Chicken	347	0.2817 ± 0.1047	0.0979 ± 0.0297	0.1906 ± 0.1046	**0.3275 ± 0.0477**
Frog	106	0.2234 ± 0.1471	0.2779 ± 0.0634	0.2112 ± 0.0712	**0.3028 ± 0.1191**
Human	381	−0.1243 ± 0.1911	**−0.0813 ± 0.0960**	−0.2018 ± 0.0870	−0.1384 ± 0.0196
Mean	-	0.2148 ± 0.0783	0.2029 ± 0.0476	0.2136 ± 0.0693	**0.2622 ± 0.0736**

**Table 3:** RMSE performance comparison under AL settings. The best result is highlighted in bold and the second-best result is underlined.

Method	Round 1	Round 2	Round 3	Round 4	*p* Value
Random	0.9465 ± 0.0169	0.9065 ± 0.0139	0.8751 ± 0.0078	0.8637 ± 0.0087	-
Entropy [34]	0.9465 ± 0.0169	0.9180 ± 0.0137	0.8944 ± 0.0146	0.8917 ± 0.0156	0.0899
CoreSet [39]	0.9465 ± 0.0169	0.8687 ± 0.0077	**0.8418 ± 0.0089**	0.8418 ± 0.0104	0.0705
ProbCover [40]	0.9465 ± 0.0169	**0.8585 ± 0.0239**	0.8546 ± 0.0200	0.8504 ± 0.0176	0.1365
TiDAL [38]	0.9465 ± 0.0169	0.8673 ± 0.0154	0.8656 ± 0.0167	0.8647 ± 0.0166	0.2938
PMAL	0.9465 ± 0.0169	0.8741 ± 0.0082	0.8439 ± 0.0083	**0.8277 ± 0.0096**	**0.0587**

**Table 4:** R^2^ performance comparison under AL settings. The best result is highlighted in bold and the second-best result is underlined.

Method	Round 1	Round 2	Round 3	Round 4	*p* Value
Random	0.0550 ± 0.0345	0.1226 ± 0.0323	0.1788 ± 0.0100	0.1968 ± 0.0121	-
Entropy [34]	0.0550 ± 0.0345	0.1188 ± 0.0215	0.1666 ± 0.0209	0.1699 ± 0.0222	0.1700
CoreSet [39]	0.0550 ± 0.0345	0.1702 ± 0.0321	0.2258 ± 0.0224	0.2323 ± 0.0212	0.0622
ProbCover [40]	0.0550 ± 0.0345	**0.2158 ± 0.0371**	0.2230 ± 0.0308	0.2326 ± 0.0239	0.1094
TiDAL [38]	0.0550 ± 0.0345	0.2087 ± 0.0277	0.2131 ± 0.0297	0.2131 ± 0.0297	0.1646
PMAL	0.0550 ± 0.0345	0.1759 ± 0.0200	**0.2263 ± 0.0166**	**0.2514 ± 0.0166**	**0.0586**

## Data Availability

The data are available in https://toxric.bioinforai.tech/ (accessed on 18 November 2022).
